# Multicenter Serologic Investigation of Influenza D Virus in Cats and Dogs, Europe, 2015–2024

**DOI:** 10.3201/eid3202.251164

**Published:** 2026-02

**Authors:** Claudia Maria Trombetta, Aurora Fiori, Alessandro Falsini, Francesco Pellegrini, Sophie Le Poder, Amit Eichenbaum, Venetia Cardona, Justine Oliva, Gilles Meyer, Nataliia Muzyka, Denys Muzyka, Emanuele Montomoli, Barbara di Martino, Mariette F. Ducatez, Gianvito Lanave, Vito Martella, Michele Camero

**Affiliations:** University of Siena, Siena, Italy (C.M. Trombetta, A. Fiori, A. Falsini, E. Montomoli); University of Bari Aldo Moro, Valenzano, Italy (F. Pellegrini, G. Lanave, V. Martella, M. Camero); Ecole Nationale Vétérinaire d’Alfort, Maisons-Alfort, France (S. Le Poder, A. Eichenbaum, V. Cardona); Université de Toulouse, Toulouse, France (J. Oliva, G. Meyer, M.F. Ducatez); National Scientific Center Institute of Experimental and Clinical Veterinary Medicine, Kharkiv, Ukraine (N. Muzyka; D. Muzyka); Linnaeus University, Kalmar, Sweden (D. Muzyka); VisMederi srl, Siena (E. Montomoli); University of Teramo, Teramo, Italy (B. di Martino); University of Veterinary Medicine, Budapest, Hungary (V. Martella)

**Keywords:** influenza D virus, influenza, viruses, respiratory infections, seroprevalence, cats, dogs, Europe, France, Italy, Ukraine

## Abstract

We conducted a multicenter study in Europe (France, Italy, and Ukraine) to assess the seroprevalence of influenza D virus (IDV) in domestic cats and dogs. Serum samples from France (2015–2018) and Italy (2023–2024) showed no IDV positivity. In Ukraine, 2.46% of dogs and 0.85% of cats tested IDV-positive in 2024.

Influenza D virus (IDV) has cattle as its primary reservoir but can occasionally spill over into other species (*1*,*2*). Recent serologic findings in dogs (Italy) and cats (China) suggest a broader host range than previously recognized (*3*,*4*). We investigated IDV seroprevalence in feline and canine samples in a multicenter study in Italy, France, and Ukraine.

In the Apulia region of Italy, we obtained serum samples from 76 domestic cats in 2023 and 56 domestic cats in 2024 collected by veterinary offices either for presurgical evaluation or routine analysis. In France, we obtained serum samples from 114 dogs and 47 cats collected during animal hospitalizations in 2015–2018 at the companion animal clinic at École Vétérinaire de Maisons-Alfort in the Ile-de-France region. Dog nasal swab (n = 41) and lung tissue (n = 24) samples originated from either a shelter or from clinics from animals with respiratory clinical signs. In Ukraine, we collected serum samples from 118 domestic cats and 122 dogs in 2020, 2023, and 2024 from veterinary clinics from the oblasts (i.e., administrative divisions) of Donetsk, Zaporizhzhia, Khmelnytska, Odesa, Kyiv, Lviv, Kharkiv, and Dnipropetrovsk (Table 1; Appendix). 

**Table 1:** Overview of samples, including country, year of collection, testing assays, IDV strains, and other information, analyzed for multicenter serologic investigation of IDV in cats and dogs, Europe, 2015–2024*

Animal species	Country	No. samples (type)	Year of collection	Assay	IDV strain	Other tests or assays	
Cat	France	47 (serum)	2015–2018	HI	D/OK	ICV HI, IAV NP ELISA	
	Italy	132 (serum)	2023–2024	HI	D/OK, D/660	None	
	Ukraine	118 (serum)	2020–2023–2024	HI	D/OK, D/660	None	
Dog	France	114 (serum), 41 (nasal swab), 24 (lung fragment)	2015–2018	HI, qRT-PCR	D/OK	ICV HI, IAV NP ELISA	
	Ukraine	122 (serum)	2020–2023–2024	HI	D/OK, D/660	IAV NP ELISA	

*HI, hemagglutination inhibition; IAV NP ELISA, influenza A virus antibodies by nucleoprotein ELISA; ICV, influenza C virus; IDV, influenza D virus, qRT-PCR, real-time reverse transcription PCR.

We tested all samples in duplicate by hemagglutination inhibition (HI) assay by using IDV strains from 2 viral lineages: strain D/bovine/Oklahoma/660/2013, D/660 lineage, and either strain D/swine/Italy/199724–3/2015 or strain D/bovine/France/5920/2014, D/OK lineage. Samples from France and Ukraine underwent further testing (Table 1)

Samples from 3/122 dogs (2.46% [95% CI 0.51%–7.02%]) and 1/118 cat (0.85% [95% CI 0.02%–4.63%]) collected in Ukraine in 2024 tested positive for D/660; the samples originated primarily from Odesa Oblast, except for 1 dog sample from Zaporizhzhia Oblast (Table 2). All samples were negative for influenza A virus by ELISA. All swab and tissue samples from France were IDV-negative by real-time reverse transcription PCR.

**Table 2:** HI titers of samples collected in 2024 in Ukraine, as part of multicenter serologic investigation of influenza D virus in cats and dogs, Europe, 2015–2024*

Sample no.	HI assay titers	
	D/660 lineage	D/OK lineage
Dog	20–20	<10
180	20–20	<10
182	20–20	<10
184	10–10	<10
Cat		
181	20–20	<10

*Titers below the detectable threshold of 10 were expressed as <10 and considered negative. HI, hemagglutination inhibition; IDV, influenza D virus.

Our findings provide evidence of IDV exposure in clinical healthy domestic cats (0.85%) and dogs (2.46%) from the Odesa and Zaporizhzhia oblasts in Ukraine, although the dogs and cats from those regions had relatively low IDV seropositivity rates and titers. Those results align with 2 recent studies on IDV circulation in dogs and cats, indicating seroprevalences of 1.2% in 2016 and 4.7% in 2023 for D/660 in dogs in southern Italy and of 2.22% in cats in northern China (*3*,*4*). In the study in China, household cats showed substantially higher exposure rates than stray cats, probably because of increased human contact, whereas the independent lifestyles of stray cats might limit exposure. 

In our study, the source of IDV infection in cats remains unclear. Although serologic testing cannot confirm active transmission or whether infection with IDV can cause disease or clinical signs, the seroprevalence in domestic cats and dogs suggests that close human–animal interactions might increase exposure risks. All the positive samples from 2024 belonged to the D/660 lineage, which was first detected in Europe in 2018 (*5*). None of these samples reacted against the D/OK lineage, which was likely replaced by D/660 lineage in Europe since 2019 (*5*).

The samples from pets in Italy and France tested negative for IDV. Because the samples in France were only tested against D/OK, results suggest that the D/OK lineage possibly was no longer circulating in France or, at least, not in the surveyed area. Factors such as urbanization and the limited presence of cattle and other susceptible species might be involved.

We also tested samples in France for influenza C virus (ICV); 2.63% of dogs tested positive, and HI titers ranged from 20 to 80, supporting previous evidence of dog susceptibility to ICV infection. An older study conducted in France during 1988–1989 found HI reactivity in dogs as high as 32% and titers ranging from 1:20 to 1:320 (*6*).

One limitation of our study is that we used a convenience collection of samples. In addition, in Italy and France we collected samples from a single geographic area. Moreover, information on age and sex of the collected animals was not available. Furthermore, we used different assays and different IDV strains and lineages for screening at the various locations. For example, for the IDV screening, we tested the samples in France only for D/OK, but we tested the samples in Italy and Ukraine for D/OK and D/660. Only the France samples were tested for ICV.

Overall, our findings suggest that household dogs and cats might be exposed to IDV and could serve as a potential source of human infection. Proactive surveillance in pets is critical to understand the changing epidemiology of IDV and to mitigate potential public health concerns. In a One Health perspective, cats and dogs are uniquely positioned to act as reservoirs for influenza virus infections in both household and rural environments.

Appendix Additional information about multicenter serologic investigation of influenza D virus in cats and dogs, Europe, 2015–2024.
