# Supplementary material for: Multicenter Serologic Investigation of Influenza D Virus in Cats and Dogs, Europe, 2015–2024
Source: Emerg Infect Dis. 2026 Feb;32(2):293–5. doi: 10.3201/eid3202.251164 (PMC12928237; doi:10.3201/eid3202.251164)
Supplement: Appendix — Additional information about multicenter serologic investigation of influenza D virus in cats and dogs, Europe, 2015–2024. [file 25-1164-Techapp-s1.pdf]

Article DOI: https://doi.org/10.3201/eid3202.251164

EID cannot ensure accessibility for supplementary materials supplied by authors. Readers who have difficulty accessing supplementary content should contact the authors for assistance.

Multicenter Serologic Investigation of Influenza D Virus in Cats and Dogs, Europe, 2015–2024

Appendix

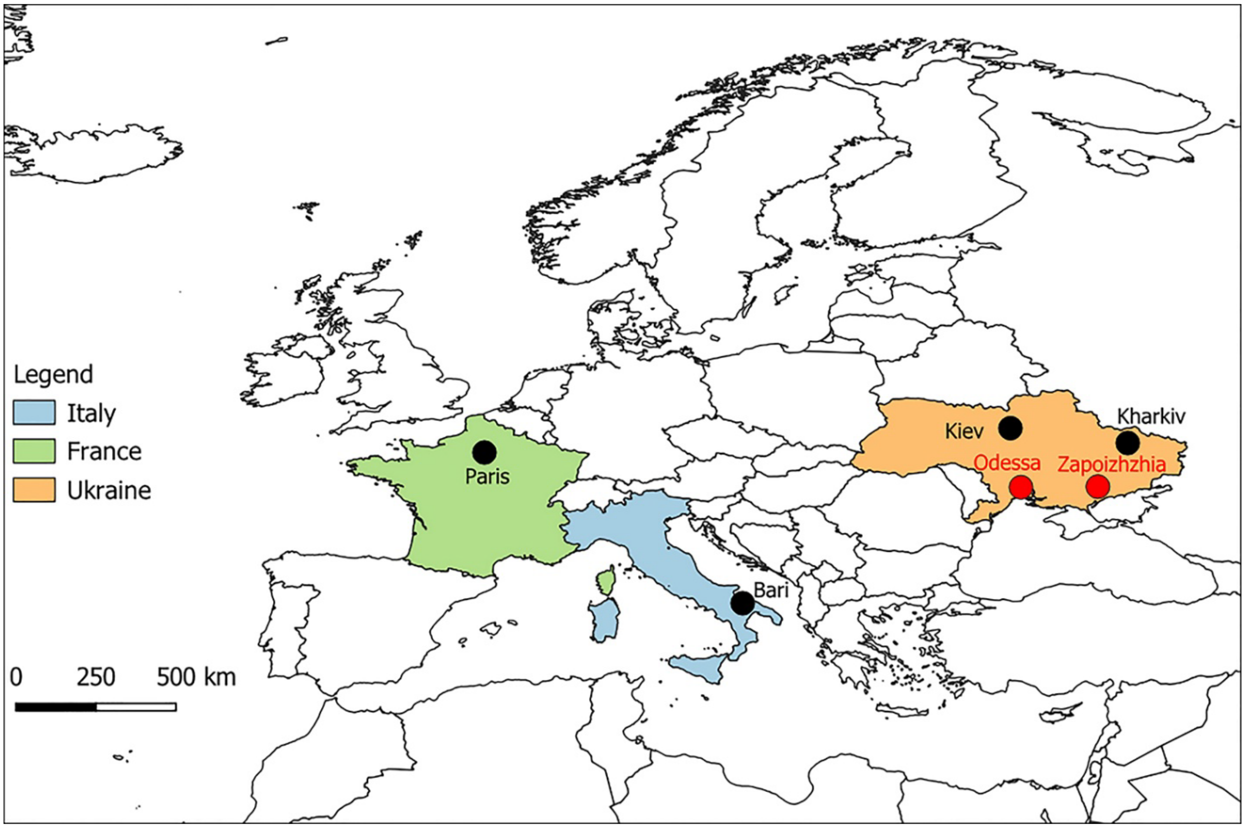

Appendix Figure. Geographic representation of the main areas across Europe where samples were collected. Red circles indicate regions where Influenza D virus–positive samples were detected.
